# Health Tract, No. 259

**Published:** 1866-09

**Authors:** 


HEALTH TRACT, No. 259.

### IMMAGINATIONS.

An English farmer became possessed with the idea that he had the Rinderpest; his family Doctor tried to laugh him out of it, this only served to confirm his vagary; he then consulted an old physician of considerable experience in human nature as well as in medicine; he made many inquiries of his patient, entered fully into the case and at lengtht sent him to an apothecary with a sealed prescription, which the man of the pestle and mortar read to the astounded patient. "This man has got the cattle plague, take him into the back yard, and shoot him on the spot, according to act of parliament." This brought the soft headed farmer to his senses, and he was a well man.

Sickness is sometimes imaginary, but in such cases it does no good to deride or to scold: so it is sometimes with what is called nervousness, it is useless to make light of it, the feeling of suffering is the same as if it were real, in such cases sympathy is oftentimes a more efficient remedy than derision or impatient epithet. "Bear ye one another's burdens" is a moral medicamentum of great efficacy. The wits of physicians are often called into requisition, and impromptu remedies are sometimes as efficacious as they are amusing. A titled lady once became possessed with the idea that a mouse had ran down her throat while she was sleeping with her mouth open; her physician seeing at once how matters stood, advised her to call next day: meanwhile he produced a mouse and arranged it in his coat sleeve so as to be made proper use of at the desired moment. With a great show of preparation he adjusted an instrument to distend the mouth, and placed a small mirror in a situation as if to reflect the image of what might be seen. "Hold on, be steady, I see the tail," and with a tremendous jerk he produced an innocent little mouse, gingerly held between thumb and finger by its caudle extremity; to the infinite gratification of his titled patient, who, placing a magnificent fee in the Doctor's hand, withdrew with a mountain-weight removed from her mind, which otherwise might have crushed it. A rich old toper imagined that a bottle was attached to his nose and that if it was broken, it would let all the blood out of his body; hence his whole time was spent in guarding his nasal appendage from harm. A rough old surgeon of great eminence was consulted, "Go to Ballylack with you," and with an appropriate action, smashed a bottle into a thousand pieces, "there's the bottle, but you see it had no blood in it." The patient's whims were humored, and the mind saved. But it is useful to observe, that it is only those who have nothing to do, persons of elegant leisure, who are cursed with these imaginary evils. Blessed is the ordainment that man shouid live by the sweat of his brow.

